# Graphene oxide-assisted non-immobilized SELEX of okdaic acid aptamer and the analytical application of aptasensor

**DOI:** 10.1038/srep21665

**Published:** 2016-02-22

**Authors:** Huajie Gu, Nuo Duan, Shijia Wu, Liling Hao, Yu Xia, Xiaoyuan Ma, Zhouping Wang

**Affiliations:** 1 State Key Laboratory of Food Science and Technology, Synergetic Innovation Center of Food Safety and Nutrition, School of Food Science and Technology, Jiangnan University, Wuxi, 214122, China

## Abstract

Okadaic acid (OA) is a low-molecular-weight marine toxin from shellfish that causes abdominal pain, vomiting and diarrhea, i.e., diarrheic shellfish poisoning. In this study, a ssDNA aptamer that specifically binds to OA with high affinity was obtained via Systematic Evolution of Ligands by Exponential Enrichment (SELEX) assisted by graphene oxide (GO). This aptamer was then applied to fabricate a novel direct competitive enzyme-linked aptamer assay (ELAA). At the optimized conditions, this ELAA method showed a low detection limit (LOD of 0.01 ng/mL), wide linear range (from 0.025 to 10 ng/mL), good recovery rate (92.86–103.34% in OA-spiked clam samples) and repeatability (RSD of 2.28–4.53%). The proposed method can be used to detect OA in seafood products with high sensitivity and can potentially be adapted for the determination of other small molecular analytes.

Okadaic acid (OA) is a low-molecular-weight lipophilic marine toxin^1^. The toxin is produced by some algae, such as Dinoflagellates (e.g., Dinophysis), and then accumulates in shellfish, such as clams, mussels, oysters and scallops, when the shellfish filter these toxin-producing algae^2^. OA strongly inhibits protein phosphatases (PPs) by binding to the PP1 and PP2A receptors and blocking their activity^3^,^4^. Once humans consume seafood contaminated with OA, the hyperphosphorylation of protein will dysregulate sodium secretion and solute permeability. This dysregulation results in the primary symptom of diarrheic shellfish poisoning (DSP), diarrhea^5^. OA was also identified to be a tumor promoter^6^. Because it is widespread, OA has been recognized as a threat to human health and the seafood industry. Therefore, the detection of OA in seafood is of great importance for food safety monitoring.

Various analytical methods for OA detection have been developed, including bioassays^7^,^8^, chemical techniques^9^,^10^ and biochemical methods^11^,^12^,^13^. The mouse bioassay (MBA) is the classic screening method for biotoxins detecting, but its drawbacks include poor reproducibility and high variability because susceptibility to biotoxins differs by mouse strains, gender, body weight, and other factors^14^,^15^,^16^. Furthermore, ethical issues are also a serious concern associated with this assay^17^. Alternatively, cytotoxicity assays have been used to detect OA. In these bioassays, morphological changes in cells are observed, or cytotoxicity is analyzed based on the colorimetric results of an Methylthiazolyldiphenyltetrazolium bromide (MTT) assay. However, this method is time-consuming because of the cell culture, and the identification of toxin category is difficult due to confusing results^18^. Chromatography coupled with fluorescence detection or mass spectrometry has been accepted as a highly selective sensitive reference method to quantify OA^2^. Other chemical techniques, such as capillary electrophoresis and surface plasmon resonance, have also been developed. However, these chemical methods invariably require expensive instruments, professional operators, and complicated pretreatment processes^19^. Thus, they are not suitable for on-site testing. Biochemical methods include protein phosphatases inhibition-based and immunology-based methods assisted by colorimetry^20^,^21^, fluorescence^22^ and electrochemistry^23^,^24^. Furthermore, relevant commercial kits have already appeared on the market. Because of the strong inhibition of OA to PPs and the high affinity of the antibody for OA, these kits are sensitive, rapid and easy to use. However, the sensitivity of protein phosphatases to OA differs by company, and the enzymatic activity is not always stable^19^. Antibody preparation relies on animal immunization, which is not practical for low-molecular-weight targets without immunogenicity. Therefore, the production process is complex and time-consuming. Furthermore, the antibody is unstable for long-term storage.

An aptamer is a single-stranded DNA (ssDNA) or RNA selected from a random oligonucleotide library via systematic evolution of ligands by exponential enrichment (SELEX)^25^. As novel molecular recognition elements, aptamers have significant advantages over antibodies, such as better affinity and specificity, wider varieties of targets, easier synthesis and modification, higher stability, and lower cost^26^,^27^. Because of these advantages, aptamers have been widely used in the fields of medical diagnosis and therapy, chemical analysis, and food safety^28^,^29^,^30^. Each round of SELEX usually consists of several steps as follows: target binding, separation between bound and unbound sequences, elution of aptamers, PCR amplification, and preparation of single-stranded aptamers^31^. The most crucial step is separating target-bound sequences from unbound sequences. Small molecule targets are usually immobilized on solid substrates, such as microplate wells^32^, beads^33^ or columns^34^ to capture affinity sequences, allowing free sequences without affinity to be washed away. However, the immobilization process may change the original conformation of the target; the steric hindrance of the immobilized target may block the binding site, and nonspecific binding to a solid substrate may lead to nonspecific sequences. To overcome these disadvantages, graphene oxide (GO) has been used in the separation step to fabricate a novel immobilization-free screening method^35^,^36^ because it can adsorb single-stranded oligonucleotides via π-π stacking^37^. GO can adsorb the unbound sequences and let the sequences bound to target free. The unbound sequences and GO can then be removed by centrifugation.

The enzyme-linked immunosorbent assay (ELISA) is one of the most common immunology-based assays and has been commercially exploited. Recently, the enzyme-linked aptamer assay (ELAA), a variation of the ELISA in which the antibody is replaced by an aptamer, has been developed^27^. As with the ELISA, the ELAA can be divided into several classes, including sandwich^38^, direct^39^ and indirect^32^ competitive assays. Because of the above-described advantages of aptamers, ELAA may gradually replace ELISA in the future.

In this study, an aptamer against OA with high affinity and specificity was screened using GO-assisted SELEX and then used to develop a novel direct competitive ELAA for the detection of OA in clams. In this direct competitive assay, a short complementary sequence instead of an enzyme-labeled toxin was used to compete with toxin present in the sample to bind to the immobilized aptamer. This proposed method offers a low detection limit, wide linear range, good recovery rate and repeatability.

## Results and Discussion

### Selection of ssDNA aptamer against OA assisted by graphene oxide

**Figure 1:**
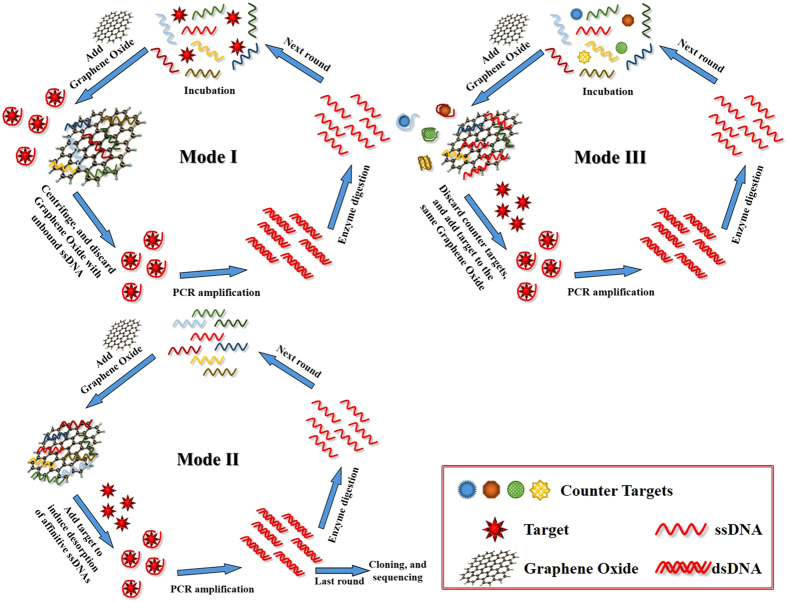
Schematic illustration of three models of GO-SELEX.

Screening several aptamers that recognize OA with high affinity and specificity from a vast random library consisting of 10^15^–10^18^ different ssDNA molecules is challenging. In our SELEX, three selection protocols were used to achieve the goal (Fig. 1). Mode I, which was previously reported^35^, was used in the early rounds because of the low ratio of affinity ssDNAs in the library. The ssDNA pools were first incubated with OA in order to generate affinity complexes. After the addition of GO, most ssDNAs adsorbed to GO via π-π stacking interactions, and only a few ssDNAs bound to OA remained in solution, which were then amplified, enzyme digested and purified for the next round. Mode II was performed in last rounds to remove weakly bound ssDNAs. After several screening rounds, the affinitive ssDNAs were enriched. When OA was added to the ssDNA pool adsorbed to GO, only the ssDNAs with strong binding ability could be induced to change conformation and detach from GO^37^. However, if the Mode II process was employed in the early rounds, target molecules may not have had a chance to attach and induce conformational change-based release due to the low number of affinitive ssDNAs adsorbed to the GO. Mode III was a counter step used to remove the non-specific ssDNAs. Four counter targets were selected, including two structural analogs, dinophysistoxin-1 (DTX-1) and dinophysistoxin-2 (DTX-2), and two main marine toxins, saxitoxin (STX) and Domoic acid (DA), which may co-exist with OA in a heterogeneous environment. In the counter GO-SELEX round, the counter target-bound ssDNAs that remained in the supernatant were eliminated by centrifugation. Subsequently, OA was added to the precipitate containing the GO-bound ssDNAs to induce desorption due to affinity-based conformational changes. This process increased the specificity while offering the same stringency of screening as Mode II.

As described in Supplementary Table S2, along with the increasing of the SELEX rounds, the conditions were modulated to increase the selective pressure. The decreasing amounts and shortening incubation time of ssDNA and OA reduced the probability of binding between ssDNA and OA. The incubation time of ssDNA and GO was lengthened to allow sufficient time for the adsorption of non-specific and weakly bound ssDNAs on GO. The three SELEX modes and increasing stringency of conditions contributed to the selection of the optimal aptamer by gradually removing non-specific and low-affinity ssDNAs.

The ssDNA pool from the thirteenth round was cloned and sequenced, resulting in 34 sequences. Eight aptamer candidates were selected for further analysis based on their sequence repetitiveness, representative secondary structures and lower predicted free energies (see Supplementary Table S3).

**Figure 2:**
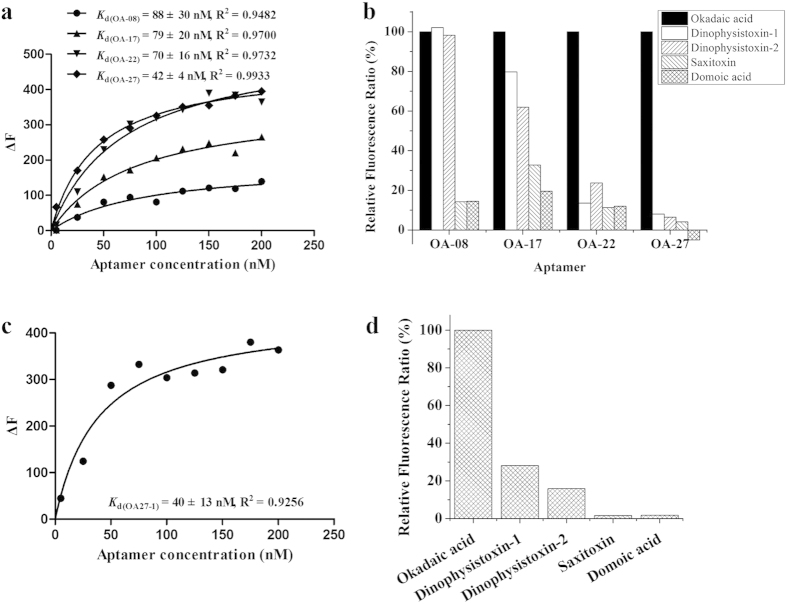
The binding affinity and specificity assays. **(a)** Binding saturation curves of selected aptamer candidates to OA. The *K*_d_ values were determined by a nonlinear regression analysis using GraphPad Prism 5.0. **(b)** Characterization of the specificity of aptamer candidates. **(c)** Binding saturation curve and *K*_d_ value of truncated aptamer OA27-1. **(d)** Characterization of the specificity of truncated aptamer OA27-1. ΔF = F −F_0_, where F and F_0_ are the fluorescence intensities at 520 nm in the presence and absence of target, respectively. Relative fluorescence ratio = ΔF_t_/ΔF_OA_, where ΔF_t_ and ΔF_OA_ are the ΔF values of each target and Okadaic acid, respectively.

To evaluate the binding affinities of the selected aptamer candidates, a dose dependent experiment was performed by incubating 1 μM OA with increasing concentrations of the synthetic sequences labeled with 5(6)- Carboxyfluorescein (FAM) (5–200 nM) in dark for 2 h. Then, the corresponding amounts of GO with the same weight ratio to ssDNAs were added and incubated in dark for 1 h. After centrifugation at 12000 r/min for 15 min, the fluorescence intensity of the supernatant containing OA-aptamer complexes was determined by a FL-7000 fluorescence spectrophotometer. Negative controls consisting of aptamer candidates and GO but without the target OA were conducted to eliminate self-desorption of ssDNAs from GO. The binding saturation curves and dissociation constants (*K*_d_) were obtained by nonlinear regression analysis of GraphPad Prism 5.0. Figure 2(a) shows the binding saturation curves. The affinity assay demonstrated that the *K*_d_ values of four aptamer candidates — OA-28, OA-17, OA-22, OA-27 — were lower than 100 nM, which showed stronger binding affinities to OA than the other four sequences. The R^2^ values ranged from 0.94 to 0.99 for the four sequences, suggesting good fits and reliably calculated *K*_d_ values.

The four aptamer candidates with lower *K*_d_ values were then further studied with a specificity test. The specificity test was conducted using a method similar to the affinity assay by incubating 150 nM aptamer candidates with 1 μM different targets (OA, DTX-1, DTX-2, STX, DA) in dark for 2 h. Negative control of each sequence was mixed with binding buffer instead of marine toxins. Subsequently, GO was added and incubated in dark for 1 h, followed by centrifuging and measuring the fluorescence intensity of the supernatant. As presented in Fig. 2(b), OA-22 and OA-27 exhibited markedly lower relative fluorescence ratio values than OA in the presence of counter targets, and aptamer OA-27 was found to be significantly more specific to target OA, whereas OA-08 and OA-17 showed obvious cross-reactivity with structure analogues DTX-1 and DTX-2. These results confirmed that OA-27 had the highest affinity and specificity, with a *K*_d_ value of 42 nM and no cross-reaction with counter targets.

The three-dimensional structure, including complementary molecular shape interaction, precise stacking of flat moieties, specific hydrogen bonding and electrostatic interaction, is crucial for target recognition and binding^25^,^40^. If the secondary structure of the truncated central region is similar to that of the entire sequence, they may have the similar affinity and specificity^36^. As shown in Supplementary Fig. S2, the stem-loop structures of position 4–14 and position 19–33 in truncated OA27-1 were the same as that of position 24–34 and position 39–53 in OA-27. Thus, the FAM-labeled truncated sequence OA27-1 was synthesized and subjected to affinity and specificity tests. Figure 2(c) shows the binding saturation curve of OA27-1, and the calculated *K*_d_ values was 40 nM, indicating a similar binding affinity to that of OA-27. The result of the specificity assay indicated that OA27-1 was less specific to OA than the entire OA-27 sequence but more specific than the other three sequences (Fig. 2(d)). Considering that shorter aptamer sequences formed simpler but sufficient structures to specifically recognize targets with high affinity^41^,^42^, OA27-1 was used as a molecular recognition element for the subsequent ELAA.

### Direct competitive ELAA

**Figure 3:**
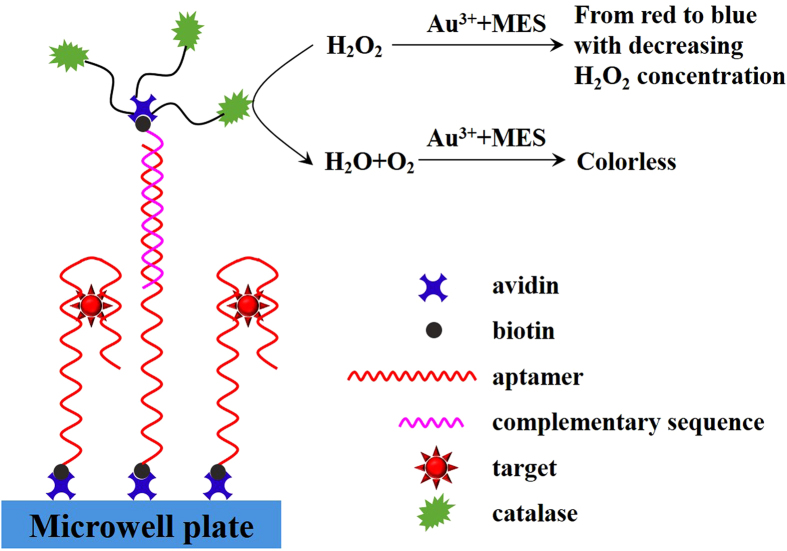
Schematic illustration of direct competitive ELAA.

Sandwich-type assays are not suitable for OA determination because OA may be too small to bind to two aptamers. Thus, a competitive assay was performed for OA detection based on the direct competition between biotinylated complementary sequence and free OA present in sample for the immobilized aptamer. As illustrated in Fig. 3, the aptamer was first fixed on the microplate through the specific binding between biotin and avidin. The biotinylated complementary sequence hybridized with the aptamer was labeled with avidin-catalase conjugate. In the absence of OA, a large amount of the catalase could consume H_2_O_2_. Low concentration of H_2_O_2_ could reduce gold trichloric acid to aggregated nanoparticles, which caused the solution to turn blue. In the present of OA, the complementary sequence was replaced, resulting in high concentration of H_2_O_2_. Then, non-aggregated gold nanoparticles were formed, and the solution was red.

#### Optimization of assay conditions

In order to develop the direct competitive ELAA, optimization of assay conditions was performed.

**Figure 4:**
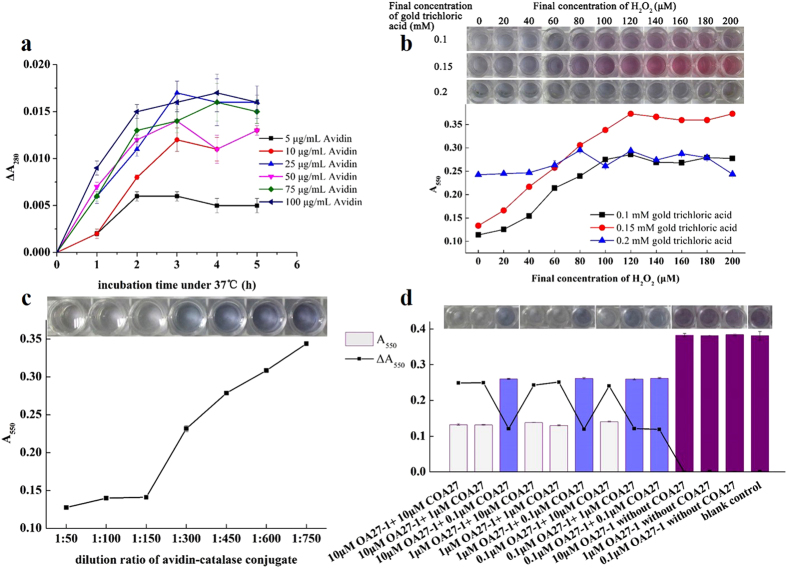
Optimization of assay conditions. **(a)** Reduced absorbance of avidin under different coating conditions, including avidin concentration and incubation time. **(b)** Absorbance at 550 nm of the reaction of different concentrations of gold trichloric acid with gradient concentrations of H_2_O_2_, and a photograph showing the color variation of the microplate. **(c)** Absorbance at 550 nm of the negative experiments using different dilution ratios of avidin-catalase conjugate, and photograph showing the color change of the microplate. **(d)** A_550_ and ΔA_550_ of the checkerboard experiment with different concentrations of OA27-1 and COA27, and a photograph showing the color change of the microplate.

As shown in Fig. 4(a), after 25 μg/mL avidin was loaded to microplate and incubated at 37 °C for 3 h, the ΔA_280_ reached a maximum, indicating that avidin coating was maximized to immobilize the biotinylated aptamer. Consequently, the optimal coating conditions were 25 μg/mL avidin and incubation at 37 °C for 3 h.

Figure 4(b) shows that 0.1 mM and 0.15 mM gold trichloric acid gradually increased the absorbance at 550 nm until the H_2_O_2_ concentration increased to 120 μM. The color correspondingly changed from colorless to purple (0.1 mM gold trichloric acid) or red (0.15 mM gold trichloric acid). The difference in the A_550_ between the maximum and minimum was larger for 0.15 mM gold trichloric acid than for 0.1 mM gold trichloric acid. The A_550_ values of 0.2 mM gold trichloric acid slightly fluctuated, and the colors were almost the same. Therefore, 0.15 mM gold trichloric acid and 120 μM H_2_O_2_ were ideal concentrations of chromogenic agents to obtain larger absorbance changes for a wider linear range.

Figure 4(c) shows that the signal intensity sharply increased as the avidin-catalase dilution ratio increased from 1:150 to 1:750. For dilution ratios of 1:50, 1:100 and 1:150, the signals were very low, indicating that catalase was sufficient to hydrolyze most H_2_O_2_, which prevented a reaction between gold trichloric acid and H_2_O_2_. Thus, the dilution ratio of 1:150 was suitable for the experiment.

Figure 4(d) shows controls, which lacked COA27, and the blank control, which lacked both OA27-1 and COA 27. These samples had the largest signal intensities because catalase was not immobilized in the well, resulting in a high concentration of H_2_O_2_. When OA27-1 and COA27 were used, the avidin-catalase conjugate was connected to COA27 via the reaction between avidin and biotin. The H_2_O_2_ concentration decreased due to catalase hydrolysis, and the magnitude of this decrease directly correlated with the change in signal intensity occurred. To obtain a calibration curve with a larger range, 1 μM OA27-1 and 1 μM COA27 were optimized to maximize the signal change.

#### Detection of OA using direct competitive ELAA

**Figure 5:**
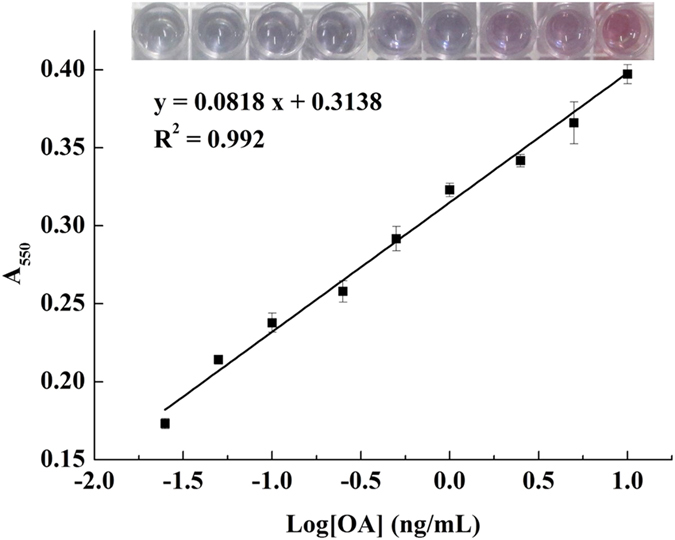
Calibration curve of the absorbance at 550 nm versus OA concentration measured by dc-ELAA.

After optimization, a direct competitive assay was carried out. Once OA was added to the reaction system, it competed with the complementary sequence for the aptamer. The more concentration of OA, the less concentration of complementary sequences, which decreased the amount of connected avidin-catalase and increased the amount of H_2_O_2_ that survived hydrolysis, resulting in different colors. Therefore, unlike the inhibition curve in other competitive assays, in this ELAA method, the signal intensity became stronger with the growing concentration of OA. In other words, the absorbance at 550 nm was directly proportional to the concentration of OA. A series of A_550_ values were measured in the presence of various concentrations of OA. As shown in Fig. 5, a strong linear correlation (R^2^ = 0.992) was obtained between the signal intensities and OA concentrations ranging from 0.025–10 ng/mL. The limit of detection (LOD) of the proposed method for OA quantification was calculated to be 0.01 ng/mL based on the equation LOD = 3SD/slope, where SD represents the standard deviation of blank samples and the slope was obtained from the calibration curve.

To validate the effectiveness of the present method, clam samples were spiked with 0.5, 1, and 10 times the FDA regulatory guidance level of OA (20 μg/100 g tissue)^43^. The spiked samples were treated and analyzed simultaneously using the dc-ELAA method and ELISA test kit. As shown in Table 1, the mean recovery rates detected by dc-ELAA and ELISA were 92.86–103.34% and 91.23–99.37%, respectively, and the relative standard deviation (RSD) values were both less than 6%. These results confirmed that dc-ELAA is as satisfactory a detection method as ELISA. Compared with other published methods for OA detection, the dc-ELAA method in the present study has a lower LOD value and wider detection range, especially at low concentrations (Table 2). Therefore, the proposed method can effectively and sensitively detect OA in samples in realistic environments.

## Conclusions

In summary, we first selected aptamers against OA as molecular recognition elements and then developed a direct competitive enzyme-linked aptamer assay method to detect OA in shellfish. The *K*_d_ values of the obtained aptamers, OA-27 and OA27-1, were slightly lower than that of the aptamer screened by Shimaa Eissa (OA34) using OA coated beads^44^, which exhibited a higher affinity for target OA. More importantly, the target in our screening process was free molecular OA, not OA coupled to beads, as described in Eissa’s study. Therefore, the aptamer in our study may be more effective in detecting samples containing OA in its native conformation. The developed dc-ELAA is novel because the absorbance signal directly correlated with OA concentrations, whereas these two parameters inversely correlated in other competitive assays. The optimized method exhibited a high sensitivity (LOD of 0.01 ng/mL), a wide detective range (from 0.025 to 10 ng/mL), and high recovery rates (92.86–103.34% in OA spiked clam samples). Given the high affinity and specificity of the selected aptamer, the established aptasensor could provide a simple and sensitive tool to detect OA in seafood products. Furthermore, this assay may be adapted for the detection of any small molecular analyte after selecting a relevant aptamer with high affinity and specificity.

## Methods

The reagents and instrumentations used in this study were listed in supplementary information.

### *In vitro* selection of the DNA aptamer assisted by graphene oxide

Three different screening processes (Fig. 1) were used to obtain aptamers that recognize OA with high affinity and specificity.

GO-SELEX Mode I: the ssDNA pool was first incubated with OA at 37 °C, and GO solution was then added. The resulting mixture was incubated at 37 °C to adsorb the free unbound ssDNAs. The mixture was centrifuged at 12000 r/min for 15 min to remove unbound ssDNAs and GO, and the ssDNAs bound to OA remaining in the supernatant were then amplified by PCR. Subsequently, the PCR products were purified and digested by lambda exonuclease to prepare ssDNAs. Finally, the ssDNAs were purified as a sub-library in the next round.

GO-SELEX Mode II: initially, the sub-library was adsorbed to GO, followed by centrifuging and washing the precipitate three times. After resuspending the GO precipitate, OA was added, and the mixture was incubated to desorb the affinitive ssDNAs. Subsequently, the mixture was centrifuged, and the supernatant containing ssDNAs bound to OA was amplified and purified. A sub-library was prepared by lambda exonuclease digestion and ethanol precipitation.

GO-SELEX Mode III (counter SELEX): the counter SELEX process was employed to eliminate nonspecific binding to analogues and other marine toxins. Two analogues, DTX-1 and DTX-2, and two main marine toxins, STX (a paralytic shellfish poison) and DA (an amnesic shellfish poison), were selected as counter targets. First, the counter targets were mixed with the ssDNA pool, and GO was then added. The supernatant containing nonspecific ssDNAs bound to counter targets was removed by centrifugation. Next, the addition of OA to the precipitate led affinitive ssDNAs to change their conformation and be released from GO. The ssDNAs bound to OA were collected by centrifugation, followed by PCR amplification, ssDNA preparation and purification.

Before each SELEX round, the ssDNA original library (see Supplementary Table S1) or sub-library dissolved in binding buffer (BB: 50 mM Tris, 150 mM NaCl, 2 mM MgCl_2_, pH 7.4) was heated at 95 °C for 5 min and cooled on ice for 15 min to achieve the best conformational structure of oligonucleotides. In each round, the ssDNA pool was incubated with targets at equal molar ratios, whereas GO and ssDNA were incubated at a mass ratio of 300:1. The recovered ssDNA pools were amplified by PCR in 20 parallel 50 μL reactions containing 0.1 mM dNTPs, 0.2 μM forward primer, 0.2 μM phosphorylated labeled reverse primer, 2 U of Taq Plus DNA polymerase and 10 μL of template each. The PCR cycling conditions were 94 °C for 5 min, followed by 20 cycles of 94 °C for 30 s, 53 °C for 30 s, 72 °C for 30 s, and a final step of 72 °C for 5 min. The PCR products were electrophoresed in 8% native polyacrylamide gel. Lambda exonuclease was used to digest phosphorylated antisense strands to prepare ssDNAs from PCR products according to the manufacturer’s instructions, and the fragments were identified by 8% denatured polyacrylamide gel containing 7 M urea. The PCR products and enzyme digestion products were all purified by phenol/chloroform extraction, recovered by ethanol precipitation and then quantified using an ND-1000 Spectrophotometer. The detailed conditions of each SELEX round, including ssDNA library amount and incubation time, were altered to increase the stringency of SELEX (see Supplementary Table S2).

After thirteen rounds of selection, the enriched aptamer pool was amplified by PCR with the unmodified primers. The purified PCR products were cloned and sequenced by Sangon Biotech Co., Ltd. (Shanghai, China). The obtained sequences were aligned using DNAMAN V6. The secondary structures and free energies (ΔG) of the sequences were predicted by RNAstructure v5.6. Subsequently, aptamer candidates were selected and synthesized with FAM labeled at the 5′ end. The binding affinity and specificity were evaluated with a fluorescence assay.

The structure of the central aptamer region without the primer binding site was predicted by RNAstructure v5.6. The truncated central sequence, whose structure was similar to that of the complete sequence, was synthesized and analyzed.

### Direct competitive enzyme-linked aptamer assay (dc-ELAA)

#### Optimization of assay conditions

Prior to the direct competitive assay, the assay conditions, including coating conditions, concentrations of chromogenic agents (gold trichloric acid and H_2_O_2_), dilution of tracer (avidin-catalase conjugate), and concentrations of capturer (aptamer) and competitor (complementary sequence) were optimized to ensure high-quality results. A gradient concentration series of avidin in carbonate buffer (pH 9.6) was prepared, and 100 μL samples were added to wells and incubated for a period of time. The coating effect was estimated based on the reduced absorbance of avidin (ΔA_280_) before and after coating. One hundred microliters of freshly prepared different concentrations of gold trichloric acid were incubated with gradient concentrations of H_2_O_2_ in MES buffer (1 mM, pH 6.5) at room temperature for 30 min. Appropriate concentrations of chromogenic agents were selected according to the absorbance change. A series of negative experiments (without target OA) were carried out using different dilution ratios of avidin-catalase conjugate in blocking agent. The A_550_ values were compared to select a suitable dilution ratio. A checkerboard method using various concentrations of aptamer (OA27-1) and complementary sequence (COA27) was employed. The absorbance of the negative experiments (without target OA) was measured, whereas different concentrations of OA27-1 and COA27 that were both labeled with avidin at the 5′ end were used. The ΔA_550_ values (A_550blank control_ −A_550sample_) were compared.

#### Pretreatment of shellfish sample

Clams were purchased from a local supermarket and served as the shellfish sample. An OA ELISA Test Kit was used to test the clams, and the results showed that the samples were not contaminated with OA. The shellfish sample was then spiked with OA. First, the tissue sample was removed from clams, and washed with deionized water. After draining the excess liquid, the sample was homogenized to a soupy texture. Then, the homogenized tissue was weighed, and corresponding amount of OA stock solution was added and mixed by homogenization. Following weighing out 1 g of mixture, 4 mL 50% methanol was added and vortexed for 5 min. The sample was centrifuged at 4000 r/min for 10 min. Subsequently, 1 mL supernatant was transferred to a new tube, and heated at 75 °C for 5 min, followed by centrifuging at 4000 r/min for 10 min again. Finally, 50 μL clear supernatant was mixed with 950 μL of Sample Extraction Buffer (from OA ELISA TEST Kit)/Methanol (90/10, V/V) as sample for detection.

#### Detection of OA by direct competitive ELAA

The 96-well microplate was first coated with 100 μL of avidin and then blocked with 200 μL of 2% BSA in PBS (10 mM Na_2_HPO_4_, 2 mM KH_2_PO_4_, 2.7 mM KCl, 137 mM NaCl, pH 7.4) for 1 h at 37 °C. After washing three times with PBST (PBS with 0.05% Tween-20), 10 μL of biotinylated aptamer in PBS was added to each well and incubated for 30 min at 37 °C. Subsequently, the microplate was washed three times with PBST, and 10 μL of biotinylated complementary sequence in PBS was loaded and allowed to incubate at 37 °C for 30 min. After washing, 100 μL of diluted avidin-catalase was added, and the mixture was incubated for 30 min at 37 °C. The microplate was washed three times, and 100 μL of standard or sample solution containing OA was added to each well of the microplate. The microplate was incubated for 1 h at 37 °C and then washed five times with PBST, once with deionized water, and air-dried. Subsequently, 100 μL of H_2_O_2_ in MES buffer was added and incubated at room temperature for 30 min. Finally, 100 μL of gold trichloric acid in MES buffer was added to each well. After 30 min of incubation at room temperature, the absorbance at 550 nm was measured using a SpectraMax M5 microplate reader.

Furthermore, an ELISA Test Kit was used to detect OA in samples. The recovery rate and RSD of the two methods were calculated and compared.

Please see the Supplementary Information about the details on determination of the optimal mass ratio of GO/ssDNA and preparation of avidin-catalase conjugate.

## Additional Information

**How to cite this article**: Gu, H. *et al.* Graphene oxide-assisted non-immobilized SELEX of okdaic acid aptamer and the analytical application of aptasensor. *Sci. Rep.* **6**, 21665; doi: 10.1038/srep21665 (2016).

## Supplementary Material

Supplementary Information

## Figures and Tables

**Table 1:** Recovery study of dc-ELAA and ELISA.

Methods	Spikedconcentration(ng/g)	Measuredconcentration(ng/g, mean ± SD)	Recovery(%, mean)	RSD(%)
dc-ELAA	100	103.34 ± 2.35	103.34	2.28
	200	196.02 ± 8.88	98.01	4.53
	1000	928.56 ± 40.87	92.86	4.40
ELISA	100	99.37 ± 1.79	99.37	1.80
	200	182.46 ± 1.23	91.23	0.68
	1000	953.50 ± 50.11	96.35	5.20

**Table 2:** Comparison of the reported methods for OA detection.

Methods	LOD	range	Reference
MTT-based cytotoxicity assay	5.0 ng/mL	N.I.	18
HPLC/ESI-MS	0.20 ng/mL	1–2000 ng/mL	45
Colorimetric PP2A inhibition assay	0.19 μg/L (PP2A from Upstate Biotechnology) 0.96 μg/L (PP2A from GTP Technology)	0.19–5.97 μg/L (PP2A from Upstate Biotechnology) 0.75–5.97 μg/L (PP2A from GTP Technology)	23
Direct competitive SPR immunosensor	2.4 μg/L	1.4–14.9 μg/L	10
Indirect competitive ELISA	0.45 ng/mL	0.3125–50 ng/mL	46
Direct competitive chemiluminescent ELISA	0.01 ng/mL	0.03–0.2 ng/mL	47
Immunochromatographic strip	5 ng/mL	N.I.	13
Aptamer based impedimetric biosensor	70 pg/mL	100 pg/mL–60 ng/mL	44
dc-ELAA	0.01 ng/mL	0.025–10 ng/mL	This study

N.I. - No information
