# Supplementary material for: Graphene oxide-assisted non-immobilized SELEX of okdaic acid aptamer and the analytical application of aptasensor
Source: Sci Rep. 2016 Feb 22;6:21665. doi: 10.1038/srep21665 (PMC4761938; doi:10.1038/srep21665)
Supplement: Supplementary Information [file srep21665-s1.pdf]

Supplementary information

Graphene oxide-assisted non-immobilized SELEX of okdaic acid aptamer and the analytical application of aptasensor

Huajie Gu, Nuo Duan, Shijia Wu, Liling Hao, Yu Xia, Xiaoyuan Ma, Zhouping Wang *

State Key Laboratory of Food Science and Technology, Synergetic Innovation Center of Food Safety and Nutrition, School of Food Science and Technology, Jiangnan University, Wuxi, 214122, China

* Corresponding author.

Tel. / Fax: +86 510 8532 6195

E-mail address: wangzp@jiangnan.edu.cn

Materials and methods

Reagents

All sequences used in this study are listed in Supplementary Table S1. The ssDNA library which consisted of a central randomized region of 40 nucleotides (nt) flanked by two 20-nt PCR primer regions was synthesized by Integrated DNA Technologies (Coralville, USA). The primers used in PCR amplification, the selected aptamers and complementary strand used in enzyme linked aptamer assay (ELAA) were obtained from Sangon Biotech Co., Ltd. (Shanghai, China).

Okadaic acid (OA), dinophysistoxin-1 (DTX-1), saxitoxin diacetate salt (STX) were purchased from Taiwan Algal Science Inc. (Taoyuan, Taiwan). Dinophysistoxin-2 (DTX-2) was purchased from National Research Council Canada-Institute for Marine Biosciences (Halifax, Canada). Domoic acid (DA) was purchased from Enzo Life Sciences AG (Lausen, Switzerland). Acrylamide/ bis-acrylamide (30% solution), avidin, catalase were purchased from Sigma-Aldrich (IA, USA). Taq Plus DNA polymerase (5 U/μL), dNTP mixture (each 25 mM), 10×PCR buffer (containing Mg^{2+}), and other electrophoresis components were purchased from Sangon Biotech Co., Ltd. (Shanghai, China). Lambda exonuclease enzyme (5000 U/mL) and 10×lambda exonuclease reaction buffer were purchased from New England BioLabs (Ipswich, MA). Crosslinker SM(PEG)24 was purchased from Thermo Scienitfic(IL, USA). Okadaic Acid (DSP) ELISA Test Kit was purchased from BIOO Scientific Corp. (TX, USA). Other chemicals and reagents were of analytical purity and purchased from Sinopharm Chemical Reagent Co., Ltd. (Shanghai, China). Graphene oxide was prepared in our own lab as previously described[1]. All solutions were prepared with Milli-Q grade water.

Instrumentation

PCR amplification was carried out in a C1000 Thermal cycler (Bio-Rad Laboratories, Inc., Hercules, CA). DNA concentration was measured by a ND-1000 Spectrophotometer (Thermo Fisher Scientific Inc., Waltham, USA). Centrifugation was performed in an Eppendorf centrifuge 5424R (Eppendorf AG., Hamburg, Germany). Fluorescence intensity was obtained using FL-7000 fluorescence spectrophotometer (Hitachi, Ltd., Tokyo, Japan). DNA was detected by polyacrylamide gel electrophoresis in Mini-PROTEAN® tetra cell system (Bio-Rad Laboratories, Inc., Hercules, CA). Gel was captured using ChemiDOC™ XRS+ system (Bio-Rad Laboratories, Inc., Hercules, CA). ELAA was performed in standard polystyrene stripwell microplate (Corning Inc., Tewksbury, USA), and absorbance of each well was measured by SpectraMax M5 microplate reader (Molecular Devices, LLC., Sunnyvale, USA).

Determination of the optimal mass ratio of GO/ssDNA

As ssDNAs with different lengths showed different binding kinetics to GO surface[2,3], it was necessary to obtain the optimized mass ratio of GO/ssDNA. Prior to GO-SELEX, the optimization was carried out to obtain an appropriate mass ratio of GO/ssDNA in order to adsorb 80-nt ssDNA on GO completely. Different amounts of GO solution were added to 8μL 10μM 80-nt ssDNA labeled with 5(6)-FAM (equivalent to 2μg). After gently mixing, the mixtures were incubated at 37 ℃ with rotation for 1h, and then centrifuged at 12000r/min for 15min. The DNA concentration and fluorescence intensity of the supernatants were measured. As shown in Supplementary Fig. S1, DNA concentration and fluorescence intensity decreased as the mass ratio of GO/ssDNA increased. When the ratio was more than 200:1, the two signals both reached baseline. Consequently, 300:1 was chosen to ensure thorough adsorption of

ssDNA on GO.

Preparation of avidin-catalase conjugate

Avidin-catalase conjugate was a tracer to catalyse the hydrolysis of H_20_2, which could reduce gold ions to gold nanoparticles in MES buffer (1 mM, pH 6.5)[4,5]. 1 mL of 1 mg/mL avidin and 4 µL of 250 mM crosslinker SM(PEG)24 in dry DMSO were mixed and incubated at room temperature for 30 min. Then, the mixture was dialyzed in 0.1 M PBS (pH 7.3) to remove the excess crosslinker. Next, 5 mg of catalase was added to the dialysis product and kept at 4 ℃ overnight. Finally, the avidin-catalase conjugate was stored at 4 ℃ until use. After the conjugation reaction, the samples were loaded in a 10% resolving gel for electrophoresis separation to confirm the success of the conjugation reaction. Supplementary Figure S3 showed in lane 3, the avidin band disappeared, catalase band reduced, and a new band with high molecular weight appeared, which indicated that the conjugation reaction succeeded.

Figures and Tables

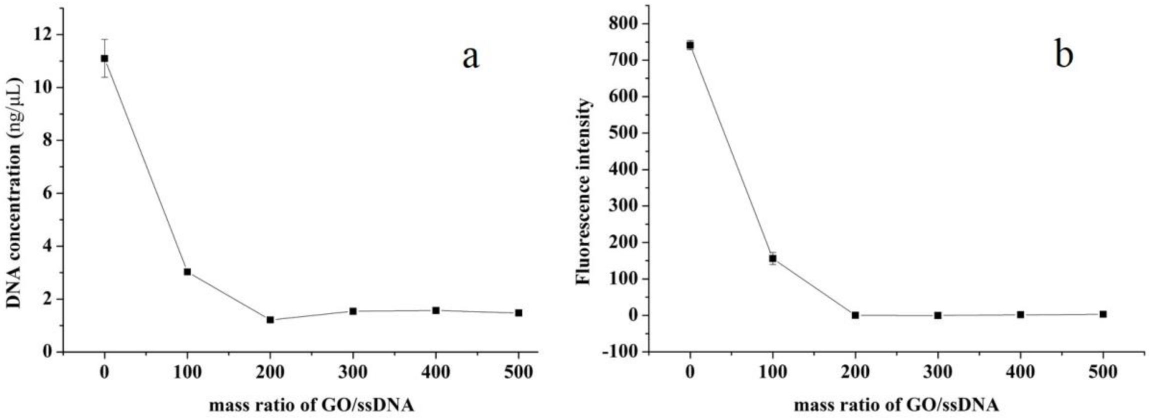

Supplementary Figure S1. Optimization of the mass ratio of GO/ssDNA. (a) DNA concentrations under different mass ratios of GO/ssDNA. (b) Fluorescence intensities under different mass ratios of GO/ssDNA.

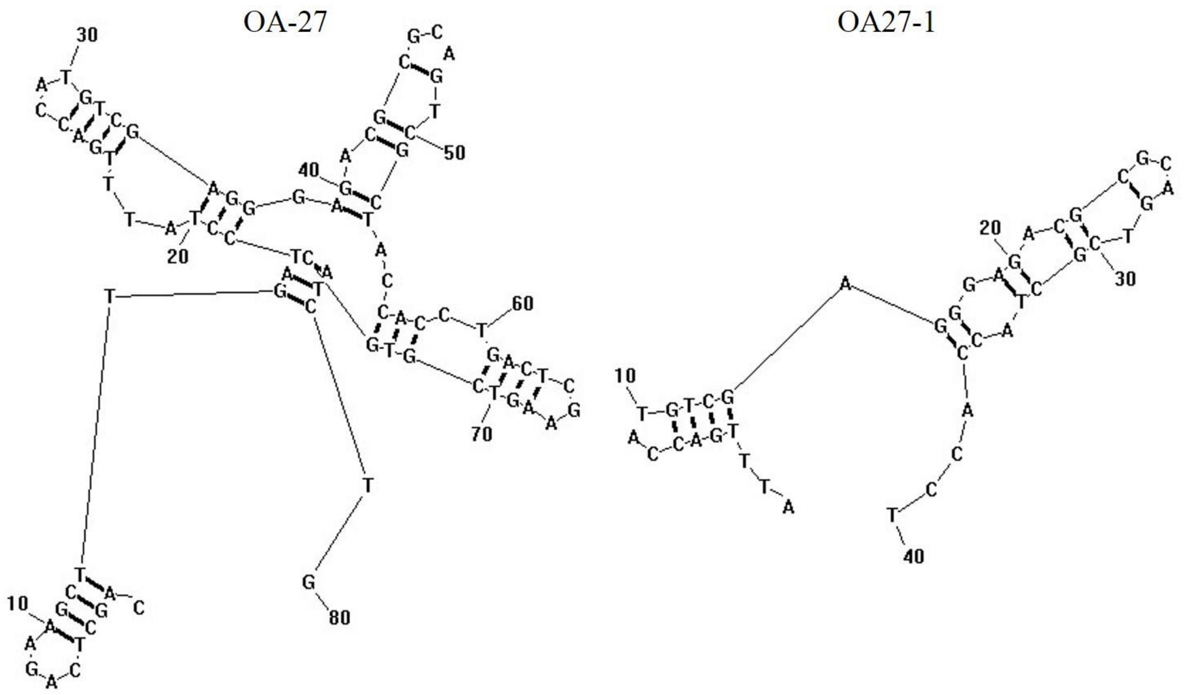

Supplementary Figure S2. Secondary structures of the selected aptamer OA-27 and its truncated sequence OA27-1 predicted by RNAstructure v5.6.

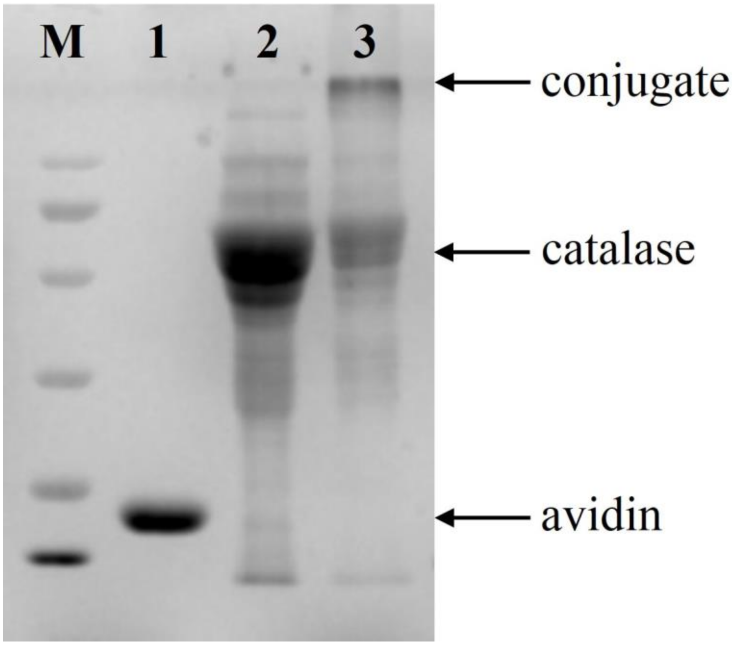

Supplementary Figure S3. SDS-PAGE image. M, standard protein marker; 1, avidin; 2, catalase; 3, avidin-catalase conjugate.

Name	Sequences
ssDNA library	5’- CAGCTCAGAA GCTTGATCCT-N_{40}- GACTCGAAGT CGTGCATCTG-3’
Forward primer (FP)	5’-CAGCTCAGAA GCTTGATCCT-3’
Reverse primer (RP)	5’-CAGATGCACG ACTTCGAGTC-3’
Phosphorylated labeled reverse primer (PFP)	5’-P-CAGATGCACG ACTTCGAGTC -3’
OA-27	5’- CAGCTCAGAA GCTTGATCCT **ATTTGACCAT GTCGAGGGAG ACGCGCAGTC GCTACCACCT** GACTCGAAGT CGTGCATCTG-3’
OA27-1	5’- **ATTTGACCAT GTCGAGGGAG ACGCGCAGTC GCTACCACCT**-3’
COA27	5’-Biotin-GATGCGTCTCCCTCG-3’

Supplementary Table S1. The sequences used in this study

Bold letters represented 40nt central region of aptamer. Underline letters represented the complementary sequence of COA27.

Round	ssDNA library（pmol/tube）	Incubation time of ssDNA library and OA（h）	Incubation time of ssDNA library and GO（h）	Counter selection targets	GO-SELEX mode
1	2000	3	1	-	Mode I:
2	200	3	1	-	Mode I:
3	200	3	1	-	Mode I:
4	200	2.5	1	-	Mode I:
5	200	2.5	1	-	Mode I:
6	200	2	1.5	STX, DA	Mode III
7	200	2	1.5	-	Mode I:
8	200	2	1.5	DTX-1<br>DTX-2	Mode III
9	200	1.5	1.5	-	Mode I:
10	100	1.5	1.5	STX, DA	Mode III
11	100	1.5	2	-	Mode II
12	100	1	2	DTX-1<br>DTX-2	Mode III
13	100	1	2	-	Mode II

Supplementary Table S2. The selection conditions in each SELEX round

The values of conditions were modulated in later rounds to enhance the stringency of the SELEX protocol.

No.	Sequences of the central randomized region	ΔG	K_d (nM)
OA-01	GCACACGAGTTACGCCCCATCGTTGTCCTTTCCCTCACGT	-10.7	125 ± 22
OA-08	AGCCCGCCAACCTACATCATCTACACTTCGTTTTAACCTT	-8.4	88 ± 30
OA-09	TAGCACACAACCCTTTGATTGGGATATCTCATCCCTCG	-11.5	127 ± 39
OA-14	ATGCTTCGTGGGACAAGAGTTGACACATCGTCGTTAGGTT	-14.0	1772 ± 543
OA-17	TTCCCCAAACTGTTGCAACCTCTGCTTTCTTTCGCTATAT	-6.5	79 ± 20
OA-21	GGATGACCGTTGCAAGCGATCCATTGTTGTTTGATCATGT	-12.5	118 ± 18
OA-22	GGCCCCGATATGATTGCTATCATTCGCATCTGCTTCACTT	-8.7	70 ± 16
OA-27	ATTTGACCATGTCGAGGGAGACGCGCAGTCGCTACCACCT	-13.5	42 ± 4

Supplementary Table S3. List of the selected aptamer candidates

Reference

1. Wu, S. *et al.* Multiplexed fluorescence resonance energy transfer aptasensor between upconversion nanoparticles and graphene oxide for the simultaneous determination of mycotoxins. *Anal. Chem.* **84,** 6263-6270 (2012).
2. He, S. *et al.* A Graphene Nanoprobe for Rapid, Sensitive, and Multicolor Fluorescent DNA Analysis. *Adv. Funct. Mater.* **20,** 453-459 (2010).
3. Wu, M., Kempaiah, R., Huang, P. J., Maheshwari, V. & Liu, J. Adsorption and desorption of DNA on graphene oxide studied by fluorescently labeled oligonucleotides. *Langmuir* **27,** 2731-2738 (2011).
4. de la Rica, R. & Stevens, M. M. Plasmonic ELISA for the ultrasensitive detection of disease biomarkers with the naked eye. *Nat. Nanotechnol.* **7,** 821-824 (2012).
5. Yuan, J. *et al.* A sensitive gold nanoparticle-based colorimetric aptasensor for Staphylococcus aureus. *Talanta* **127,** 163-168 (2014).
